# Diseases of Women

**Published:** 1889-10

**Authors:** 


**Diseases of Women: A Manual of Non-Surgical Gynecology, designed especially for the use of Students and General Practitioners.** By F. H. Davenport, A. B., M. D., Assistant in Gynecology, Harvard Medical School; Assistant Surgeon to the Free Hospital for Women; Physician to the Department of Gynecology, Boston Dispensary, with numerous illustrations. Small octavo; pp. xiv.—317. Philadelphia: Lea Brothers & Co. 1889.

When we first glanced at the title of this book, we were inclined to regard it with disfavor, because of the fact that there were too many books written on Gynecology; and, again, because we regarded it of doubtful propriety for junior teachers to enter the field of authorship; and, still again, because we are opposed to manuals as a *class*, and as a rule. The larger treatises, and the most excellent work of Mundê on Minor Surgical Gynecology, together with the transactions of the special societies and the journalistic literature, would seem to amply supply the present needs of workers in this field. But it is well not to establish hard and fast rules on this, or, for that matter, upon any other subject.

A more careful examination of the work before us compels us, in all candor, to admit that it possesses considerable merit, and that its teachings in the main are healthful and wise. What the author says on the subject of the hot water douche is a plain and excellent exposition of that important method of treatment, and may be read with profit by all physicians who treat gynecological cases.

A number of useless instruments are illustrated, among which are four cuts of Jennison's uterine repositor or sound. Now, it would appear that we are already too far from Egypt and the Sphynx to admit such instruments to our armamentaria, or to waste valuable time in writing about them in our text-books. We are opposed to the use of any sound as a repositor; or, for that matter, to any sort of a repositor; they are not only absolutely useless, but are capable of doing positive harm; but of all such instruments (?), we should place Jennison's the lowest in the scale. We have had one in our possession since 1878, and we are happy to add that we have never used it. A uterus that cannot be reposited without other means than a sound or repositor, had better be left to itself, until, if the case be a proper one for an operation, a surgeon is employed who has the pelvic and abdominal instinct. This is neither the time nor place to discuss the reposition of a displaced womb, but we have felt called upon to say this much, lest some young member of the profession would follow blindly the conclusion that all the instruments illustrated in text-books are useful, and are even needed to practice gynecology.

If all the useless instruments that have been foisted upon an unwary profession could be expurgated, the world would be better off; but what excuse can there be for authors, who are likewise teachers, to perpetrate such anomalies and perpetuate such error?

There is, as we have said at the outset, much to commend in this little book, though it may be a question of taste for a junior to make haste to write a book and to dedicate it to his senior, who is, himself, but little in advance of the author, either in years or experience. The writing of books should be left, for the most part, to men who have accumulated sufficient experience to warrant their posing as authors, and to justify their attempt to speak from the chair; and then they may properly be dedicated to still other men who have risen to supreme prominence, and have left a heritage of valuable knowledge for their successors.

The famous publishers have, as might be expected, made a handsome volume.

